# Repurposed Edaravone, Metformin, and Perampanel as a Potential Treatment for Hypoxia–Ischemia Encephalopathy: An In Vitro Study

**DOI:** 10.3390/biomedicines10123043

**Published:** 2022-11-25

**Authors:** Daniela Silva, Ruben Rocha, Ana Salomé Correia, Bárbara Mota, Maria Dulce Madeira, Nuno Vale, Armando Cardoso

**Affiliations:** 1 Unit of Anatomy, Department of Biomedicine, Faculty of Medicine, University of Porto, Alameda Professor Hernâni Monteiro, 4200-319 Porto, Portugal; 2 NeuroGen Research Group, Center for Health Technology and Services Research (CINTESIS), Rua Doutor Plácido da Costa, 4200-450 Porto, Portugal; 3 Serviço de Pediatria, Centro Hospitalar Universitário de São João, Alameda Professor Hernâni Monteiro, 4200-319 Porto, Portugal; 4 OncoPharma Research Group, Center for Health Technology and Services Research (CINTESIS), Rua Doutor Plácido da Costa, 4200-450 Porto, Portugal; 5 CINTESIS@RISE, Faculty of Medicine, University of Porto, Alameda Professor Hernâni Monteiro, 4200-319 Porto, Portugal; 6 Department of Community Medicine, Information and Health Decision Sciences (MEDCIDS), Faculty of Medicine, University of Porto, Rua Doutor Plácido da Costa, 4200-450 Porto, Portugal

**Keywords:** hypoxia–ischemia, edaravone, perampanel, metformin, OGD, ROS, overexcitation

## Abstract

Hypoxia–ischemia encephalopathy results from the interruption of oxygen delivery and blood flow to the brain. In the developing brain, it can lead to a brain injury, which is associated with high mortality rates and comorbidities. The hippocampus is one of the brain regions that may be affected by hypoxia–ischemia with consequences on cognition. Unfortunately, clinically approved therapeutics are still scarce and limited. Therefore, in this study, we aimed to test three repurposed drugs with good pharmacological properties to evaluate if they can revert, or at least attenuate, the deleterious effects of hypoxia–ischemia in an in vitro model. Edaravone, perampanel, and metformin are used for the treatment of stroke and amyotrophic lateral sclerosis, some forms of epileptic status, and diabetes type 2, respectively. Through cell viability assays, morphology analysis, and detection of reactive oxygen species (ROS) production, in two different cell lines (HT-22 and SH-SY5Y), we found that edaravone and low concentrations of perampanel are able to attenuate cell damage induced by hypoxia and oxygen-glucose deprivation. Metformin did not attenuate hypoxic-induced events, at least in the initial phase. Among these repurposed drugs, edaravone emerged as the most efficient in the attenuation of events induced by hypoxia–ischemia, and the safest, since it did not exhibit significant cytotoxicity, even in high concentrations, and induced a decrease in ROS. Our results also reinforce the view that ROS and overexcitation play an important role in the pathophysiology of hypoxia–ischemia brain injury.

## 1. Introduction

Oxygen and glucose are both essential for normal brain activity. Hypoxia–ischemia results from the combination of a lack of oxygen and blood flow interruption, and, during development, it can lead to brain injury, clinically manifested as encephalopathy and seizures. These conditions are very prevalent in neonates, particularly preterm ones, being the leading cause of neonatal deaths. They are also commonly associated with important neurological disabilities in those that survive [1,2].

Brain injury due to hypoxia–ischemia is an evolving process that can be divided into three periods [3]. The first one starts immediately after the insult, lasts approximately 6 h, and corresponds to the primary energy failure phase. In this phase, due to the decreased supply of oxygen and glucose, cells switch to anaerobic metabolism, increasing lactate production and decreasing adenosine triphosphate (ATP) production [4]. Consequently, membrane ion pumps start to fail and lead to sodium and calcium accumulation within the cells, thus releasing, from degrading structures, excitatory neurotransmitters, such as glutamate [3,5]. Glutamate is a neurotransmitter that stimulates different neuro-glial receptors, namely N-methyl-D-aspartate (NMDA) and α-amino-3-hydroxy-5-methyl-4-isoxazole propionic acid (AMPA) receptors. When in excessive amounts, it promotes a cascade response that eventually leads to excitotoxicity and reactive oxygen and nitrogen species production [6]. In the secondary energy failure phase (6 to 72 h after the insult), reperfusion is restored, which can intensify cell injury despite limiting brain damage. In the meantime, excitotoxic neurotransmitters and free radicals continue to be released, and reactive oxygen species (ROS), as well as other reactive species, are produced, thus leading to aggravation of mitochondrial dysfunction, cell death, and induction of brain self-inflammatory response [4,7]. Eventually, lesions begin to heal or settle into a chronic inflammation phase. Different mechanisms may occur during this tertiary phase (lasting months to years), including epigenetics and inflammatory changes that may induce cell death, repair, or remodeling [2,8].

**Scheme 1:**
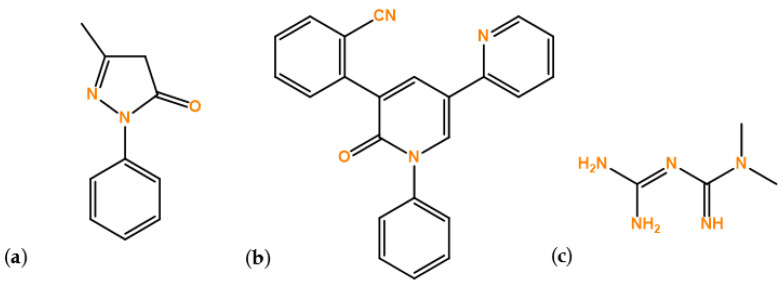
Structure of the repurposed drugs used in this study: (**a**) Edaravone, (**b**) Perampanel, and (**c**) Metformin.

Overall, neonatal hypoxia–ischemia brain injury evolves over time, which creates a window of treatment opportunity. There are different treatment approaches targeting the critical times of the different phases. Namely, for the first phase, it makes sense to try neuroprotective glutamate receptor blockers and free radical scavenger drugs [3]. After the initial phase, it is sensible to focus on anti-inflammatory, neuroprotective, and nerve regenerating drugs in order to restrain injury and promote healing. However, at present, therapeutic interventions that are clinically approved for the treatment of neonatal hypoxia–ischemia brain injury are still very limited [1]. Using repurposed drugs is potentially one way to progress faster and achieve efficient treatments. Among the repurposed drugs, we selected edaravone, perampanel, and metformin (Scheme 1).

Edaravone is a free radical scavenger [9], already approved for the treatment of amyotrophic lateral sclerosis and stroke [10]. It plays an important role not only in quenching free radicals and inhibiting lipid peroxidation [11], but also in exerting protective effects, such as anti-oxidative and pro-inflammatory responses [12].

Perampanel is an antagonist of the AMPA glutamate receptors and, therefore, is involved in inhibiting neuronal overexcitation, which may lead to neuronal protection. It has shown positive effects on seizure control in children and adults [13,14].

Metformin has been widely used to treat diabetes type 2 and is being investigated for the treatment of metabolic syndrome [15]. Its primary pharmacological activities (antioxidant and anti-inflammatory properties) can be mainly mediated by the activation of AMP-activated protein kinase (AMPK), which subsequently may modulate oxidative stress, prevent mitochondrial damage, and enhance angiogenesis [16,17]. Overall, metformin is potentially neuroprotective.

Due to their properties, these drugs have been intensely investigated for preventing neuronal damage and treating brain disorders. For instance, edaravone has already been studied for its potential to decrease neuronal deficits consequent to traumatic brain injury [18], cerebral ischemia [10,19,20], and even in a hypoxia–ischemia model [21]. Metformin also has been vastly investigated in stroke, ischemia, and dementia [17]. However, until now, there are insufficient studies that have focused on the effects of these drugs and their effects on hypoxia–ischemia brain injury.

Hence, in this work, we aimed to repurpose edaravone, perampanel, and metformin drugs in a cell-induced hypoxia–ischemia model. Using cell viability as well as ROS production, we first sought to focus on these drugs’ effects on two different cell lines, hippocampal HT-22 cells and neuroblastoma cells. Secondly, we wanted to test their potential beneficial effects on two different models of hypoxia, one using only a hypoxic atmosphere and the other using oxygen–glucose deprivation. Drugs were tested in several concentrations and times of exposure.

## 2. Materials and Methods

### 2.1. Materials

Dulbecco’s Modified Eagle’s Medium (DMEM), fetal bovine serum (FBS), and penicillin–streptomycin mixture were obtained from Millipore Sigma (Merck KGaA, Darmstadt, Germany). Dulbecco’s Modified Eagle’s Medium with no glucose (cat. no. 11966025) was obtained from Gibco (Thermo Fisher Scientific, Inc, Waltham, MA, USA). Thiazolyl blue tetrazolium bromide (MTT; cat. no. M5655), neutral red solution (NR; cat. no. N2889), sulforhodamine B (SRB; cat. no. S1402), 2, 7- dichlorofluorescein diacetate (DCFH-DA; cat. no. D6883), edaravone (cat. no. M70800), and metformin (cat. no. 317240) were obtained from Sigma–Aldrich (Merck, Algés, Portugal). Perampanel (cat. no. 23003) was obtained from Cayman (Tallinn, Estonia).

### 2.2. Cell lines and Cell Culture

The immortalized mouse hippocampal HT-22 cell line was kindly offered by Mitochondria and Neurodegenerative Disorders, CNC group. Human SH-SY5Y neuroblastoma cells were obtained from ATCC (American Type Culture Collection, Manassas, VA, USA). Both cell lines were maintained, according to recommendations, at 37 °C and 5% CO_2_, in DMEM medium supplemented with 10% FBS and 1% of an antibiotic mixture (penicillin and streptomycin). The medium was changed at least twice a week and trypsinized once a week, and it was only made when cells reached 80% or more confluence. Cells were seeded, in 96-well plates, at a density of 1 × 10^4^ cells/mL for HT-22 cells and at 1 × 10^5^ cells/mL for SH-SY5Y cells. After seeding, cells were allowed to adhere for 24 h, before exposure to the different treatments.

### 2.3. Cells Treatment

Edaravone and perampanel were dissolved in DMSO (0.1% in cell culture medium) and metformin in sterilized water (1% in cell culture medium). The three drugs were tested in cells with concentrations ranging from 0.1 to 100 µM. All treatments were performed for 6, 24, 48, and 72 h after cell attachment. For each drug, control wells were added with 0.1% of DMSO or 1% of sterilized water.

### 2.4. Oxygen Glucose Deprivation and Hypoxia Models

In oxygen–glucose deprivation (OGD) and hypoxia experiments, twin plates were created, and the induction model was performed as previously described [22,23]. For hypoxia, one twin plate was placed in a hypoxia incubator chamber (StemCell cat no.27310), with a 2% O_2_, 10% CO_2,_ and 88% N_2_ atmosphere, while the other twin plate was placed in normoxia (21% O_2_). Similarly, in OGD experiments, one twin plate was placed in the hypoxia incubator chamber, while the other twin plate was placed in normoxia. However, in this model, the plate submitted to hypoxia was cultured in DMEM-free glucose medium.

### 2.5. Cell Morphology Assessments

Cell morphology was assessed using a Leica DMI6000 B automated microscope (Wetzlar, Germany). After all treatments, cell morphology was examined and images were captured using Leica LAS X imaging software v3.7.4 (Leica Microsystems, Wetzlar, Germany).

### 2.6. Cell Viability Assays

At the end of the incubation periods, cellular viability was assessed using MTT, SRB, and NR assays. For the MTT assay, the cell medium was removed, and 100 µL of MTT solution (0.5 mg/mL in PBS) was added to each well, followed by 3 h of incubation protected from light. Then, the solution was aspirated and DMSO (100 µL/well) was added to solubilize formazan crystals. Absorbance was then measured at 570 nm, using an automated microplate reader (Tecan Infinite M200, Tecan Group Ltd., Männedorf, Switzerland). To perform the SRB protocol, the cell medium was removed, and cells were firstly washed with PBS solution. After that, cells were fixed with 10% cold trichloroacetic acid for 30 min and subsequently stained, protected from light, with 200 µL/well of SRB for 1 h. Afterwards, the plate was washed with tap water several times to remove excess dye. Finally, the dye bound to the proteins was dissolved with Tris-NaOH solution (10 mM). Absorbance was measured using a microplate reader with a wavelength of 540 nm (Tecan Infinite M200, Tecan Group Ltd., Männedorf, Switzerland). For the NR assay, 100 µL of the NR medium (1:100 in culture medium) was added to each well after the cell medium removal. After 3 h of incubation, cells were washed with 150 µL of PBS, and 150 µL of NR destain solution was added to each well. Afterward, absorbance at 540 nm was measured by using the microplate reader (Tecan Infinite M200, Tecan Group Ltd., Männedorf, Switzerland).

### 2.7. ROS Evaluation

Intracellular ROS production was measured using the fluorescent dye, DCFH-DA. Prior to drug exposure, cells were incubated for 30 min with 100 µL of DCFH-DA diluted in 1000× culture media. At the incubation end, the solution was removed, and cells were incubated with the respective drugs. Then, fluorescence was detected after the 6, 24, and 48 h treatment periods using a fluorescence plate reader (SpectraMax Gemini EM Microplate Reader, Molecular Devices, San Jose, California), with filters at 485 nm excitation and 530 nm emission.

### 2.8. Statistical and Data Analyses

GraphPad Prism 8 was used to perform statistical analysis and design graphs. The results are presented as the mean ± SEM of three independent experiments. Statistical analyses between control and treatment conditions were achieved with one-way ANOVA test. Statistical significance was considered when *p*-value < 0.05, being indicated in graphs as *, **, ***, and **** representing *p* < 0.05, *p* < 0.01, *p* < 0.001, and *p* < 0.0001, respectively.

## 3. Results

### 3.1. Effects of Edaravone, Perampanel, and Metformin on Cell Viability of HT-22 Cells

#### 3.1.1. HT-22 Cell Viability, Evaluated by MTT Assay, after 6, 24, 48, and 72 h of Treatment

**Figure 1:**
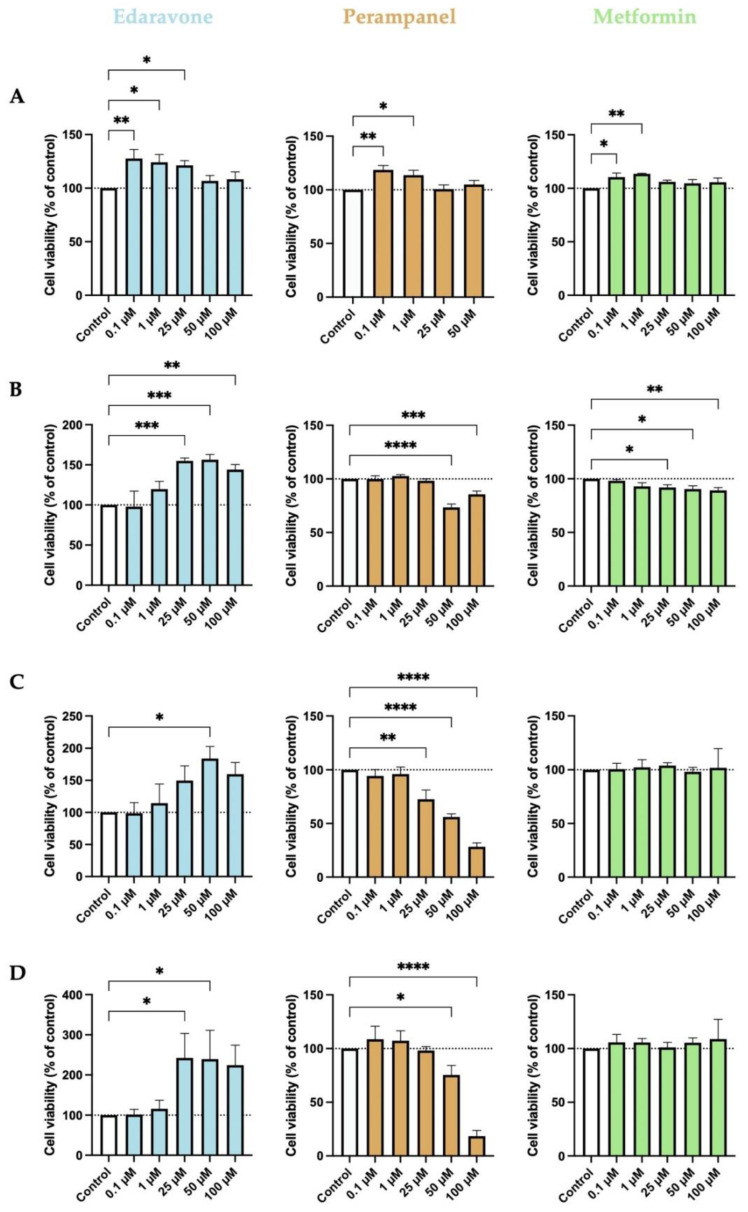
Effects of edaravone, perampanel, and metformin on the viability of HT-22 cells, at different time points of exposure. (**A**) 6-h treatment; (**B**) 24-h treatment; (**C**) 48-h treatment; (**D**) 72-h treatment. Cell viability was assessed by using the MTT assay. Results are expressed as the percentage of cell viability relative to the respective untreated control cells. *, **, ***, and **** indicate *p* < 0.05, *p* < 0.01, *p* < 0.001, and *p* < 0.0001, respectively, when compared to control. All data are presented as the mean + SEM of three independent experiments.

Firstly, we investigated the effects of increasing concentrations, ranging from 0.1 to 100 µM, of edaravone, perampanel, and metformin on HT-22 cells treated for 6, 24, 48, or 72 h. The results are expressed as the percentage of viable cells after 6 (Figure 1A), 24 (Figure 1B), 48 (Figure 1C), or 72 (Figure 1D) hours of treatment.

Our results revealed that the effects of the drugs tested varied as a function of drug concentration and duration of treatment. There was a tendency for edaravone to increase cell viability at all time points when compared to the control. Specifically, after 6 h of treatment, the lower concentrations (0.1, 1, and 25 µM) significantly increased cell viability, an effect that was not apparent when higher concentrations were used. However, after more prolonged treatment periods, particularly after 24 and 48 h, cell viability was significantly increased when the higher concentrations (25, 50, and 100 µM) were used. Relative to perampanel, only the treatment of 6 h with the two lower concentrations significantly increased cell viability. More prolonged treatments had either no effect on cell viability when the lower concentrations were used or significantly decreased cell viability when the higher concentrations were employed. When in lower concentrations (0.1 and 1 µM), metformin significantly increased cell viability only after 6 h of treatment, but it had no effect after more prolonged treatments. A higher concentration of metformin (0.25 to 100 µM) significantly decreased cell viability after 24 h of treatment but had no effect afterward.

The period of 48 h is the most used in this type of experiment since the drug has time to act, and yet there are no major changes in its composition [24]. As expected, 24- and 48-h treatments were the ones that showed better drug activities. Therefore, hereafter, we used 48-h treatment for the main tests.

#### 3.1.2. HT-22 Cell Viability, Evaluated by MTT, SRB and NR Assays, after 48 h of Treatment

**Figure 2:**
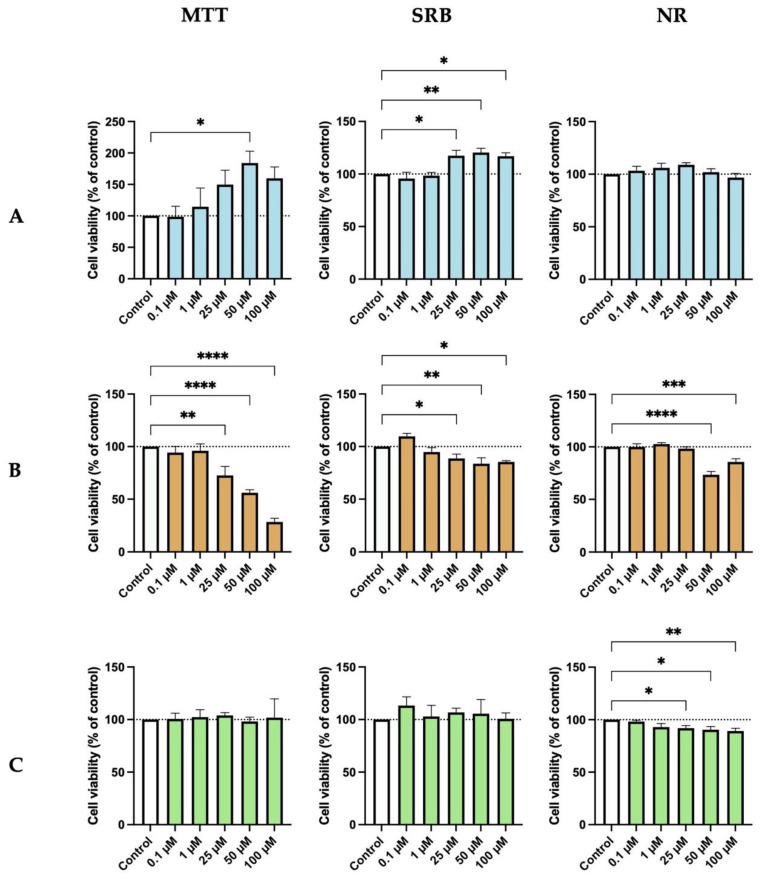
Effects of (**A**) Edaravone, (**B**) Perampanel, and (**C**) Metformin treatment on HT-22 cells viability, evaluated by three different assays. Protein content and cell viability were assessed by SRB (middle panel) and MTT and NR (left and right panel) assays, after 48 h of exposure. Results are expressed as the percentage of cell viability relative to the respective untreated control cells. *, **, ***, and **** indicate *p* < 0.05, *p* < 0.01, *p* < 0.001, and *p* < 0.0001, respectively, when compared to control. All data are presented as the mean + SEM of three independent experiments.

We proceed to evaluate whether the assay method influenced the effects of edaravone, perampanel, and metformin on HT-22 cell viability. Thus, we estimated HT-22 cell survival after 48 h of treatment with increasing concentrations of the drugs, using different indirect viability assay methods (MTT, SRB, and NR). The results show that not all assays measure cell viability at equal sensitivity. After 48 h of treatment, edaravone increased cell viability, particularly at high concentrations (50–100 µM), as shown by the MTT and SRB assays, but not by the NR assay (Figure 2A). In contrast, the effect of high concentrations (25–100 µM) of perampanel in decreasing cell viability was apparent using all assays. However, the MTT assay seemed more sensitive in detecting changes between drug concentrations (Figure 2B). Relative to metformin, the results were also similar for MTT, SRB, and NR methods (Figure 2C). However, with this drug, only the NR assay was able to demonstrate a slight, although significant, decrease in cell viability at concentrations higher than 25 µM. Overall, the MTT assay seems to be a more sensitive method to detect small changes for edaravone and perampanel and the NR assay for metformin.

#### 3.1.3. Morphological Analysis of HT-22 Cells after 48 h of Treatment with Increasing Concentrations of Edaravone, Perampanel, and Metformin

**Figure 3:**
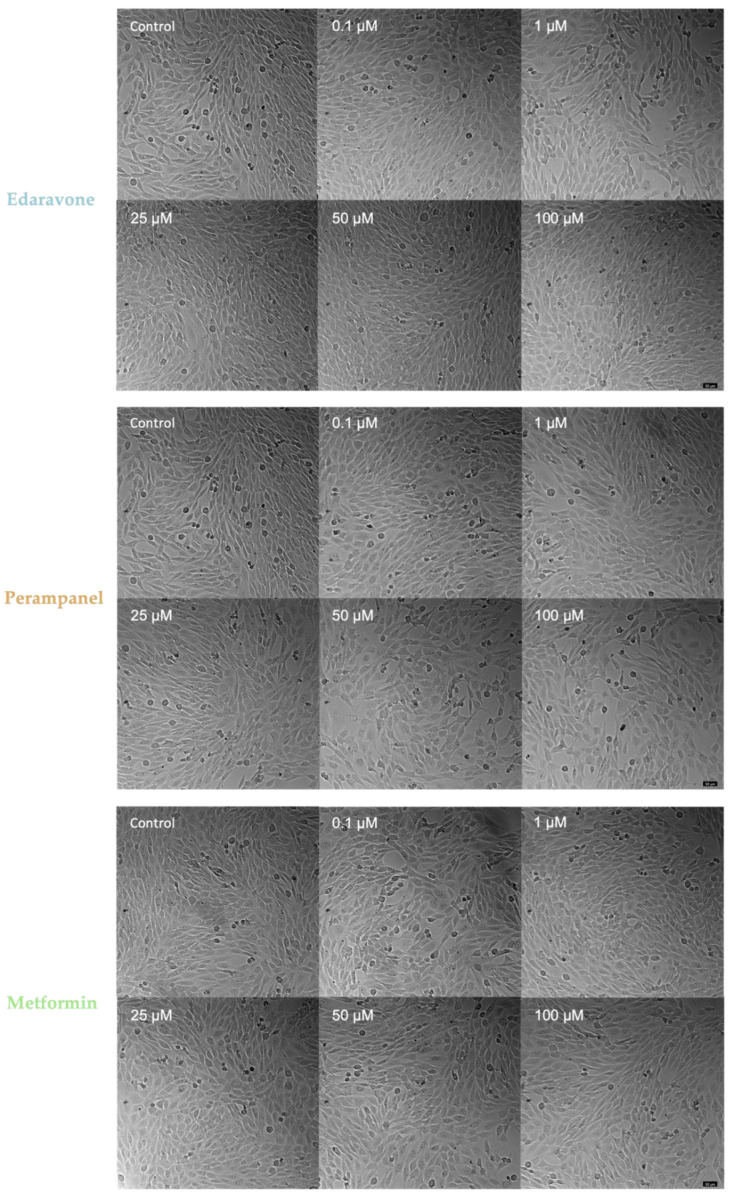
Microscopic representative images of HT-22 control cells and cells after 48-h exposure to concentrations ranging from 0.1 to 100 µM of edaravone, perampanel, and metformin. Images of cell morphology were obtained on a Leica microscope equipped with Leica LAS X imaging software v3.7.4 (Leica Microsystems, Wetzlar, Germany) from three independent experiments. Scale bar: 50 µm.

As shown in Figure 3, cells submitted to drug treatments display a normal and healthy morphology, except for those treated with higher concentrations of perampanel. After 48 h of treatment with high concentrations of perampanel, HT-22 cells were fewer, compared to the control, indicating a decrease in cell viability. However, treatment with higher concentrations of edaravone (25–100 µM) was associated with a visible increase in cell density. Lastly, treatments with metformin did not appear to induce changes in cell morphology or cell density. Globally, this morphological analysis corroborates the results obtained with MTT, SRB, and NR assay methods, reinforcing that MTT is the most representative viability assay.

### 3.2. Effects of Edaravone, Perampanel and Metformin on Viability of SH-SY5Y Cells

**Figure 4:**
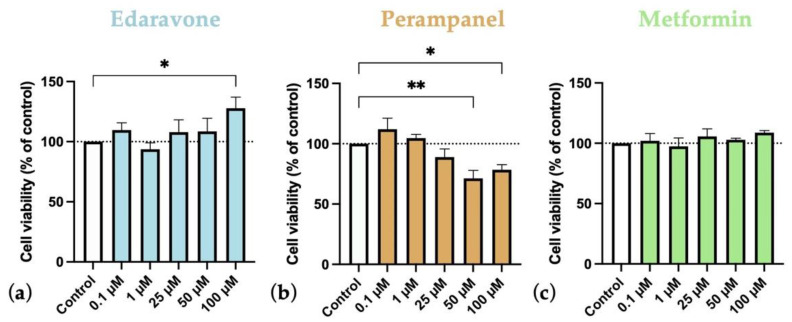
Effects of (**a**) Edaravone, (**b**) Perampanel, and (**c**) Metformin treatment for 48 h on SH-SY5Y cells viability. Cell viability was assessed by MTT assay. Results are expressed as the percentage of cell viability relative to the respective untreated control cells. * and ** indicate *p* < 0.05 and *p* < 0.01, respectively, when compared to control. All data are presented as the mean + SEM of three independent experiments.

To complement our results of edaravone, perampanel, and metformin on HT-22 cells, we also tested the same drugs on another cell line, widely used in experimental neurological studies, the SH-SY5Y neuroblastoma cell line. The cytotoxic effects detected after 48 h of treatment using the MTT assay were globally similar to those observed in HT-22 cells (Figure 4). There was a tendency for edaravone to increase cell viability at higher concentrations, but the differences were significant relative to the respective controls only at concentrations of 100 µM. Similar to HT-22 cells, perampanel also significantly decreased cell viability at higher concentrations (50–100 µM), compared to the respective control. Again, and similar to HT-22 cells, treatment with different concentrations of metformin in this cell line did not result in any significant variation in cell viability.

#### Morphological Analysis of SH-SY5Y Cells after 48 h of Treatment with Increasing Concentrations of Edaravone, Perampanel, and Metformin

**Figure 5:**
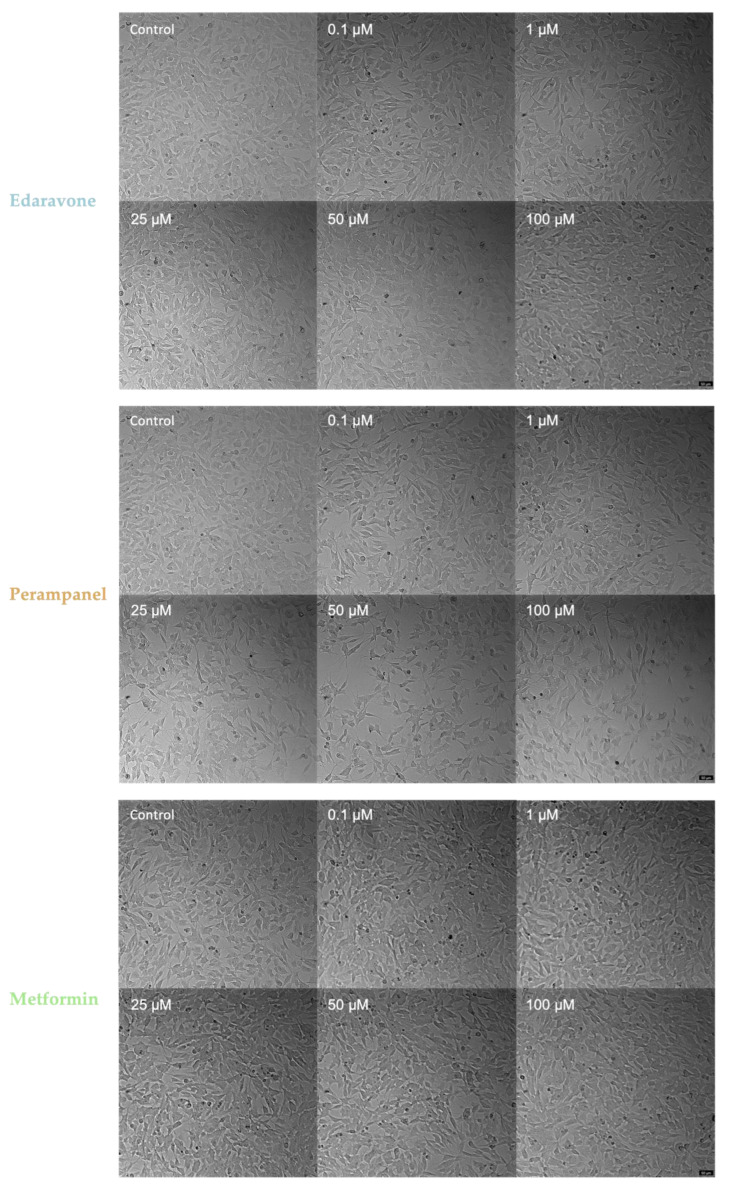
Microscopic representative images of SH-SY5Y control cells and SH-SY5Y cells exposed for 48 h to concentrations, ranging from 0.1 to 100 µM, of edaravone, perampanel, and metformin. Images of cell morphology were obtained using a Leica microscope equipped with Leica LAS X imaging software v3.7.4 (Leica Microsystems, Wetzlar, Germany) from three independent experiments. Scale bar: 50 µm.

The analysis of the morphology of SH-SY5Y cells (Figure 5) corroborates the cell viability results obtained (Figure 4). Cell density appears to be higher in edaravone-treated cells than controls, but cell morphology is apparently unchanged. Moreover, when in high concentrations, perampanel seems to be cytotoxic because it appears to decrease cell density. Metformin treatments did not significantly alter cell density or morphology. Overall, the morphological analyses are in accordance with data obtained on the viability of SH-SY5Y cells, evaluated using the MTT assay.

### 3.3. Effects of Hypoxia on HT-22 and SH-SY5Y Cells

We also analyzed treatment effects with increasing concentrations of edaravone, perampanel, and metformin, for 48 h, on both HT-22 and SH-SY5Y cells, maintained for 48 h, under hypoxia conditions. The cell viability results, estimated using the MTT assay, and cell morphology were compared with data obtained from cells of the same lines maintained under a normoxia environment.

#### 3.3.1. HT-22 Cells under Hypoxia Conditions

**Figure 6:**
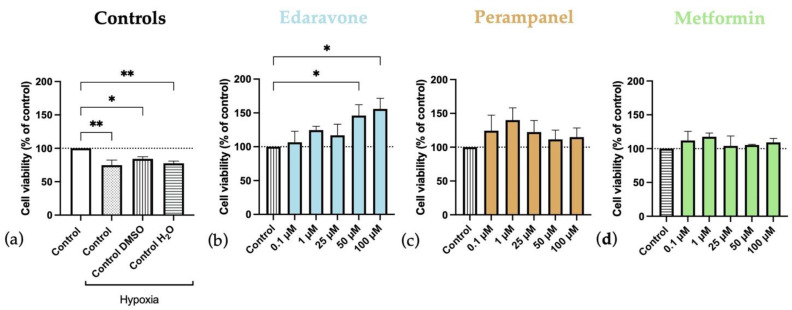
Effects of hypoxia, and edaravone, perampanel, and metformin treatments on HT-22 cells viability. Hypoxia and drugs were applied simultaneously for 48 h. (**a**) Controls graph, comparing controls/vehicles under hypoxic conditions to normoxia ones. (**b**) Edaravone, (**c**) Perampanel, and (**d**) Metformin treatments, while under hypoxia. Cell viability was evaluated by using the MTT assay. Results are expressed as the percentage of cell viability relative to respective untreated hypoxia control cells. * and ** indicate *p* < 0.05 and *p* < 0.01, respectively, when compared to the respective control under hypoxia conditions. All data are presented as the mean + SEM of three independent experiments.

**Figure 7:**
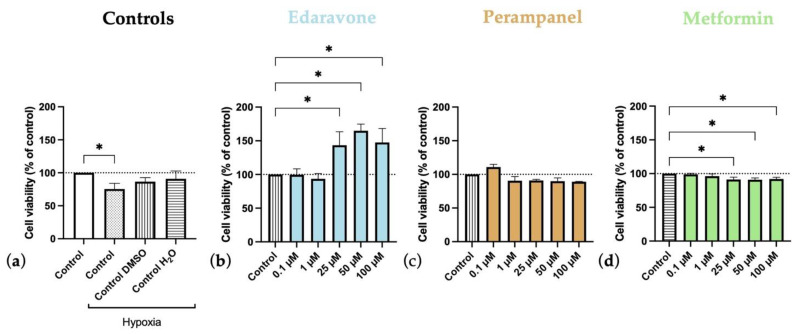
Effects of 24 h of hypoxia followed by 24-h exposure to edaravone, perampanel, and metformin on HT-22 cells viability. (**a**) Controls graph, comparing controls/vehicles in hypoxic conditions to normoxia ones. (**b**) Edaravone, (**c**) Perampanel, and (**d**) Metformin treatments, after 24 h under hypoxia. Cell viability was assessed by MTT assay. Results are expressed as the percentage of cell viability relative to the respective untreated hypoxia control cells in (**b**–**d**). * *p* < 0.05 when compared to hypoxia control. All data are presented as the mean + SEM of three independent experiments.

Our results showed that hypoxia, by itself, reduced HT-22 cell viability (Figure 6 and Figure 7). After 48 h of hypoxia (Figure 6), all controls under hypoxia showed a significant reduction in cell viability compared to the normoxia control (Figure 6a). After 48 h of treatment, on HT-22 cells under hypoxia with edaravone, there was an increase in cell viability that reached significant levels in cells treated with higher concentrations (50–100 µM) (Figure 6b). There was a similar trend for cells treated with perampanel (Figure 6c) and metformin (Figure 6d), particularly at lower concentrations (0.1–1 µM), but the differences did not reach statistically significant levels.

In another experiment delineated to analyze the second phase of hypoxia–ischemia brain injury and correlate with the injury in humans (first, the injury happens, and only after, the treatment is started), we applied the same drugs for 24 h after only 24 h of hypoxia (Figure 7). It was observed that 24-h hypoxia tends to decrease cell viability, albeit only significantly for hypoxia-control but not for the other vehicle controls (Figure 7a). Interestingly, edaravone significantly increased cell viability, even when administered at concentrations ranging from 25 to 100 µM (Figure 7b). Conversely, both perampanel (Figure 7c) and metformin (Figure 7d) treatments had no beneficial effects on cell viability. Metformin even significantly decreased cell viability when applied for 24 h after 24 h of hypoxia.

**Figure 8:**
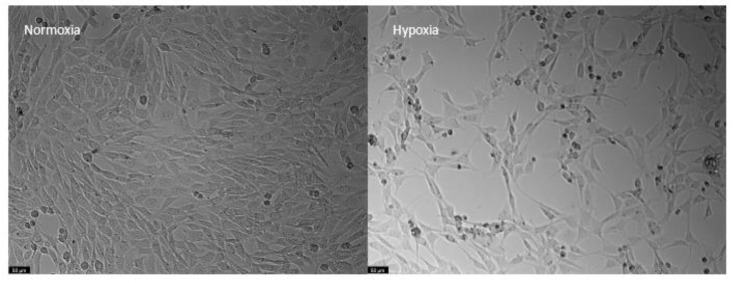
Representative images of HT-22 cells submitted to normoxia (**left**) and hypoxia (**right**) conditions for 48 h. Images of cell morphology were obtained using a Leica microscope equipped with Leica LAS X imaging software v3.7.4 (Leica Microsystems, Wetzlar, Germany). Scale bar: 50 µm.

Moreover, after 48 h of hypoxia, it was clearly visible that there were both morphological and cell density differences between cells in normoxia and hypoxia. Specifically, the cell density was lower in those submitted to hypoxia, and these cells displayed a more rounded shape (Figure 8).

#### 3.3.2. Morphological Analysis of HT-22 Cells after 48 h of Treatment with Edaravone, Perampanel, and Metformin under Hypoxia Submission for 48 h

**Figure 9:**
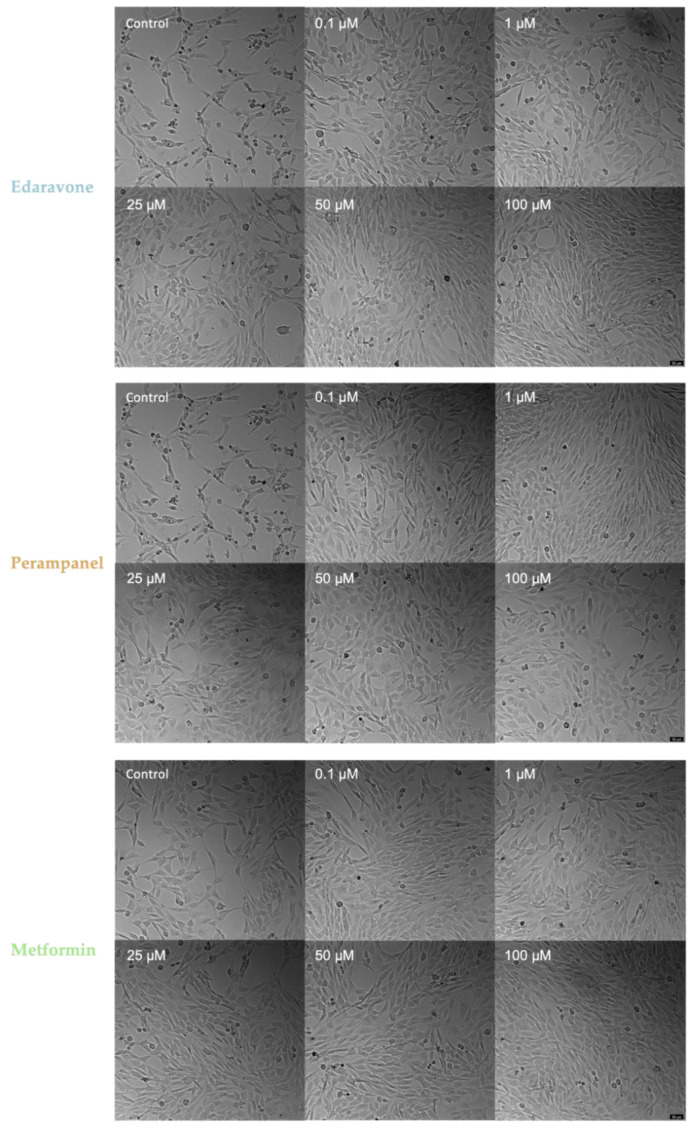
Microscopic representative images of HT-22 hypoxia control cells and HT-22 cells exposed for 48 h to hypoxia and drug concentrations, ranging from 0.1 to 100 µM, of edaravone, perampanel, and metformin. Images of cell morphology were obtained using a Leica microscope equipped with Leica LAS X imaging software v3.7.4 (Leica Microsystems, Wetzlar, Germany) from three independent experiments. Scale bar: 50 µm.

As shown globally in Figure 9, treated cells displayed a higher cell density and a more normal cell morphology. Namely, when compared to the respective hypoxia controls, edaravone and perampanel higher (50–100 µM) and lower concentrations (0.1–1 µM), respectively, promoted cell density increase and a healthier morphology appearance. These morphological results corroborate cell viability results described in Figure 6. On the other hand, the hypoxia cells treated with metformin appeared to increase cell density, which is not as visible and accentuated in the cell viability plot in Figure 6.

#### 3.3.3. SH-SY5Y under Hypoxia Conditions

**Figure 10:**
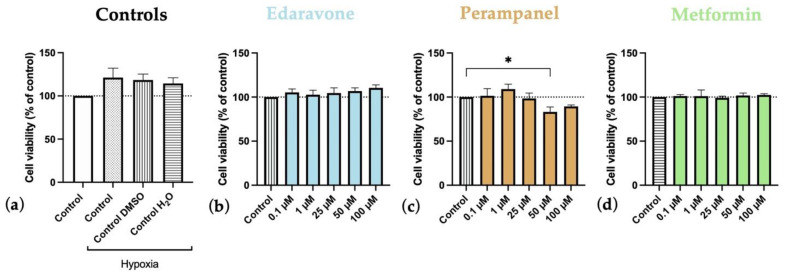
Effects of hypoxia, and edaravone, perampanel, and metformin treatments on SH-SY5Y cells viability. Hypoxia and drugs were applied simultaneously for 48 h. (**a**) Controls graph, comparing controls/vehicles in hypoxic conditions to normoxia ones. (**b**) Edaravone, (**c**) Perampanel, and (**d**) Metformin treatments, while in hypoxia. Cell viability was assessed by MTT assay, to each treatment after exposure. Results are expressed in the percentage of cell viability relative to untreated hypoxia-control cells and submitted to hypoxia in (**b**–**d**). * indicate *p* < 0.05, when compared to hypoxia-control. All data are presented as the mean + SEM of three independent experiments.

**Figure 11:**
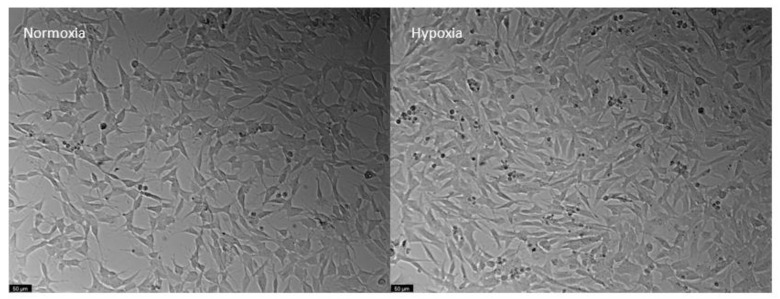
Representative images of SH-SY5Y cells submitted to normoxia (**left**) and hypoxia (**right**) conditions for 48 h. Images of cell morphology were obtained using Leica microscope equipped with Leica LAS X imaging software v3.7.4 (Leica Microsystems, Wetzlar, Germany). Scale bar: 50 µm.

The response to hypoxia of SH-SY5Y cells was different from that observed for HT-22 cells. Specifically, hypoxia did not reduce cell viability, and it even showed a trend towards increasing it, albeit not significantly, as can be seen in the control cell viability graph (Figure 10a) and in the respective representative images (Figure 11). Consequently, drug treatment (Figure 10) had no major effects on cell viability, except for 50 µM of perampanel, which significantly reduced cell viability. Regarding cell morphology, in hypoxia conditions, they appear to be as expected, pyramidal; however, it is visible that some are more rounded. These results suggest that SH-SY5Y can not only survive but even thrive in a hypoxic environment.

### 3.4. Effects of Oxygen-Glucose Deprivation on HT-22 Cells

**Figure 12:**
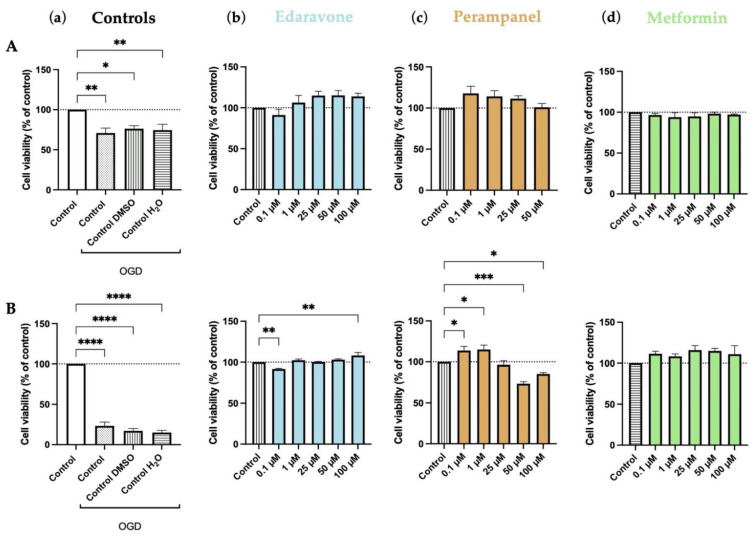
Effects of oxygen–glucose deprivation (OGD) and edaravone, perampanel, and metformin treatments for 6 (**A**) and 48 (**B**) hours on HT-22 cells viability. (**a**) Controls graph, comparing controls/vehicles in OGD conditions to standard medium. (**b**) Edaravone, (**c**) Perampanel, and (**d**) Metformin treatments, while in OGD conditions. Cell viability was assessed by MTT assay. Results are expressed as the percentage of cell viability relative to untreated control cells submitted to OGD. *, **, *** and **** indicate *p* < 0.05, *p* < 0.01, and *p* < 0.001, respectively, when compared to the respective control. All data are presented as the mean + SEM of three independent experiments.

OGD represents a severe challenge for cells, mimicking hypoxia–ischemia insults. Our results show that OGD significantly decreased the viability of HT-22 cells in all control groups, both after 6 (Figure 12A) and 48 (Figure 12B) hours of deprivation. As expected, the decrease was more severe after 48 h than after 6 h of deprivation.

During the 6-h period, edaravone and perampanel showed a trend to increase cell viability, compared to the respective OGD-treated control. However, only in the 48-h period did the differences achieve significant levels in some concentrations (100 µM for edaravone and 0.1–1 µM for perampanel; Figure 12). As previously shown, perampanel at higher concentrations reduced cell viability. Metformin did not significantly alter the viability of HT-22 cells under OGD conditions (Figure 12).

**Figure 13:**
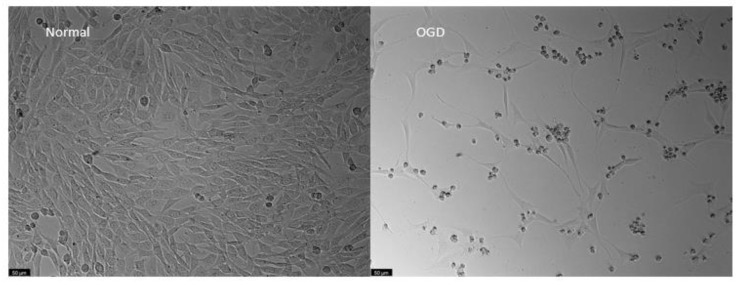
Representative images of HT-22 cells morphology, both under normal or Oxygen-Glucose Deprivation conditions during 48 h. Images of cell morphology were obtained using a Leica microscope equipped with Leica LAS X imaging software v3.7.4 (Leica Microsystems, Wetzlar, Germany). Scale bar: 50 µm.

The morphology of cells submitted to OGD was altered. Cells showed a smaller and more rounded shape, with longer prolongations than controls. Their density was also clearly reduced (Figure 13).

#### Morphological Analysis of HT-22 Cells after 6 and 48 h of Treatment with Edaravone, Perampanel, and Metformin under Oxygen Glucose Deprivation Submission

**Figure 14:**
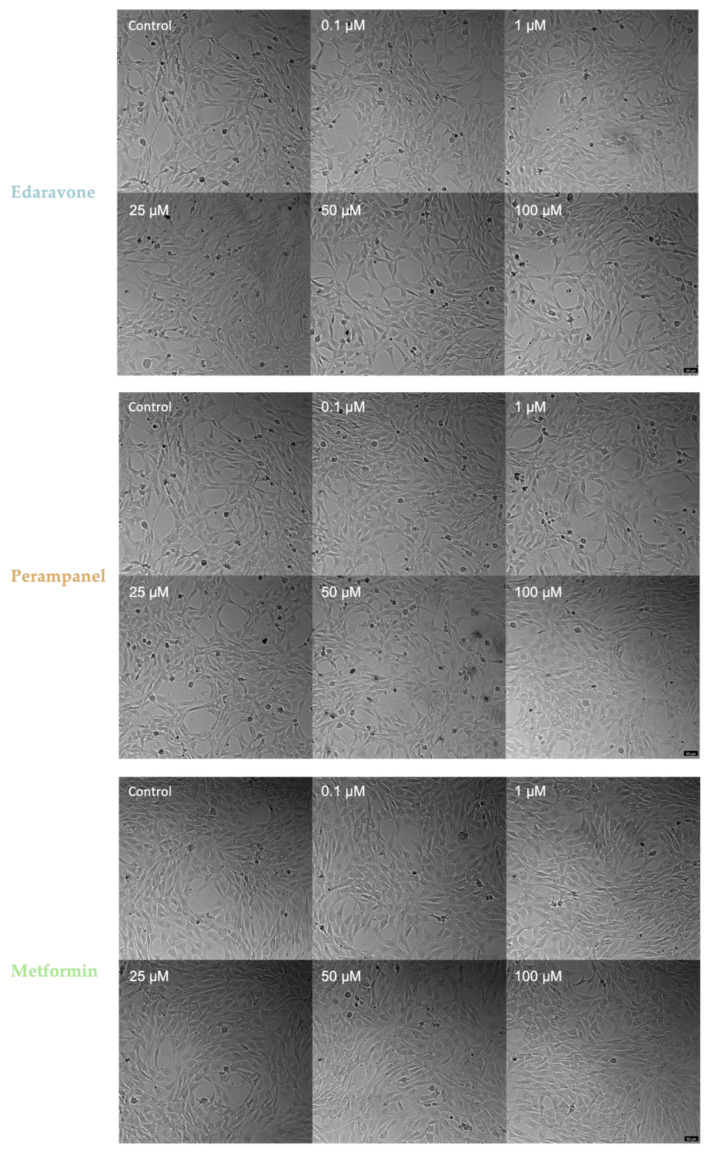
Microscopic representative images of HT-22 oxygen–glucose deprivation (OGD) control cells and HT-22 cells exposed for 6 h to OGD and concentrations, ranging from 0.1 to 100 µM, of edaravone, perampanel, and metformin. Images of cell morphology were obtained using a Leica microscope equipped with Leica LAS X imaging software v3.7.4 (Leica Microsystems, Wetzlar, Germany) from three independent experiments. Scale bar: 50 µm.

**Figure 15:**
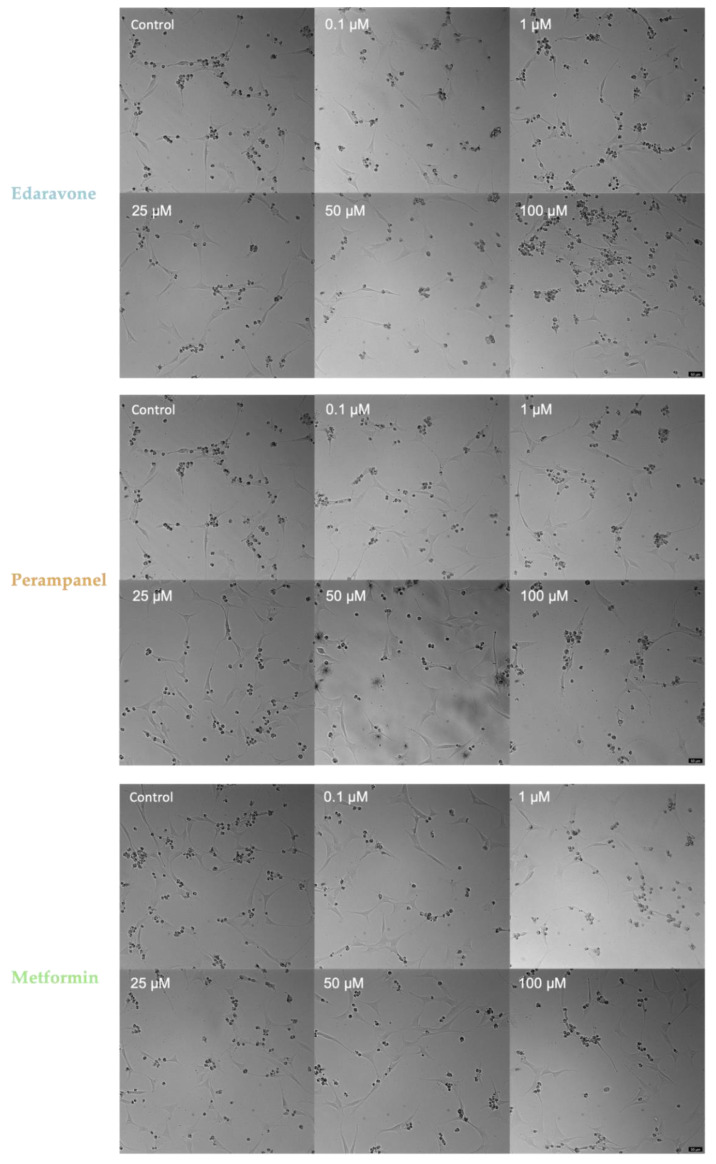
Microscopic representative images of HT-22 oxygen–glucose deprivation (OGD) control cells and HT-22 cells exposed for 48 h to OGD and concentrations, ranging from 0.1 to 100 µM, of edaravone, perampanel, and metformin. Images of cell morphology were obtained using a Leica microscope equipped with Leica LAS X imaging software v3.7.4 (Leica Microsystems, Wetzlar, Germany) from three independent experiments. Scale bar: 50 µm.

Figure 14 and Figure 15 show treated HT-22 cells morphology that were subjected to OGD for 6 and 48 h, respectively. The cells subjected to 6 h of OGD appear to undergo morphological changes, although these are not as prominent as those observed from the extensive injury caused by 48 h of OGD. In the 6-h treatment images, slight changes in perampanel cell density were observed, with an explicit increase of 0.1 µM, which is in accordance with the graphs in Figure 12A.

Despite the results from Figure 12B, there were no major changes, especially in morphology (Figure 15), between the different drug concentrations and the respective controls. However, the representative image of 100 µM edaravone treatment showed a greater number of rounded cells, which could be the reason for the statistical significance in Figure 12B. Finally, regarding morphological analysis, the individual administered drugs could not recover the cells to a healthy and normal morphology when submitted to an OGD insult.

### 3.5. Reactive Oxygen Species Measured on HT-22 Cells Submitted to OGD and Treated with Edaravone, Perampanel, and Metformin

Reactive oxygen species can be estimated and detected using DCFH-DA dye, which becomes fluorescent with ROS generation. This experiment was performed with time points of 6, 24, and 48 h.

**Figure 16:**
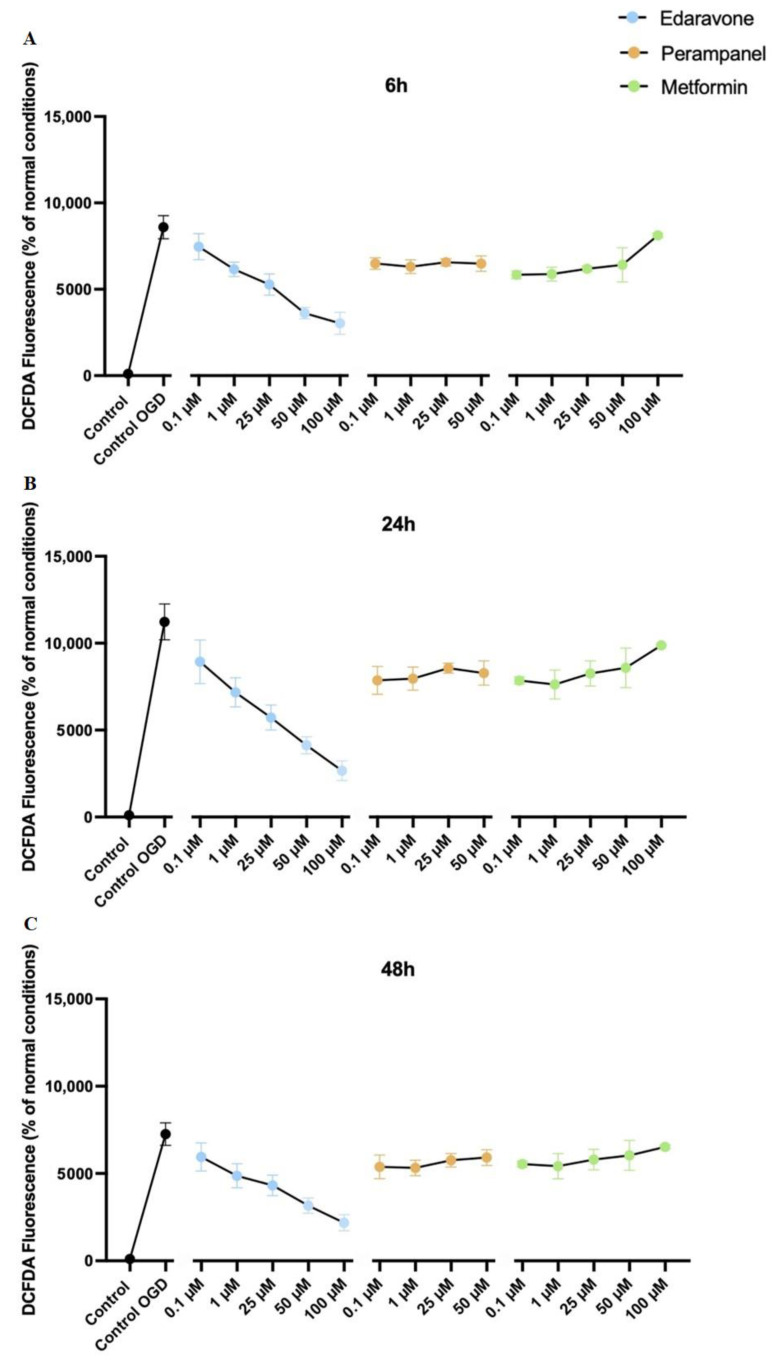
Protective efficacy of edaravone, perampanel, and metformin in HT-22 cells exposed to oxygen–glucose deprivation (OGD) over 6 (**A**), 24 (**B**), and 48 (**C**) hours. DCFDA fluorescence (dye of ROS generation) change percentage (%) of the different concentrations of edaravone, perampanel, and metformin, exposed to OGD. Results are expressed as the percentage of DCFDA fluorescence relative to control cells, in normal conditions. Results were assessed by fluorescence plate reader (SpectraMax Gemini EM Microplate Reader, Molecular Devices, San Jose, California), 485 nm excitation and 530 nm emission. Data are presented as the mean ± SEM of three independent experiments.

As expected, ROS production was increased, at all different time points, in OGD conditions. Notably, treatment of HT-22 cells submitted to OGD with the three drugs decreased ROS production, when compared to the control group. However, the decrease was greater with edaravone, where we observed significantly lower levels of ROS when cells were treated with higher concentrations of edaravone. This result reinforces the view that edaravone may protect cells submitted to hypoxic insults. Perampanel and metformin decreased ROS production when compared to the control. However, as the concentration increases for both of them, there is also an increase in ROS production, indicating that higher concentrations may induce cytotoxic effects. (Figure 16).

**Figure 17:**
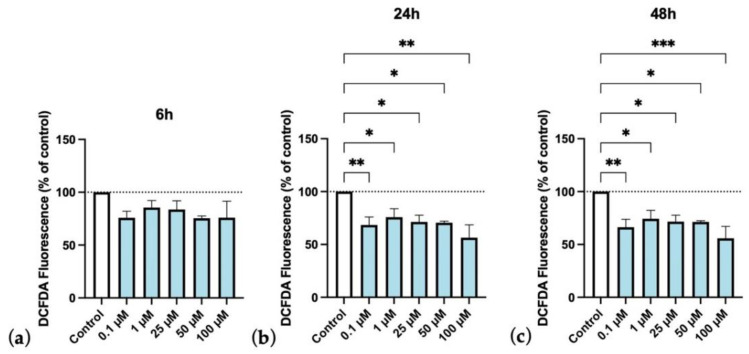
Protective efficacy of edaravone on HT-22 cells exposed to oxygen–glucose deprivation (OGD), over time (6, 24, and 48 h, (**a**), (**b**), and (**c**), respectively). DCFDA fluorescence (dye of ROS generation) change percentage (%) of the different concentrations of edaravone exposed to OGD conditions over control. Results are expressed as the percentage of DCFDA fluorescence relative to untreated control cells in OGD. Results were assessed by fluorescence plate reader (SpectraMax Gemini EM Microplate Reader, Molecular Devices, San Jose, California), 485 nm excitation and 530 nm emission. All data are presented as the mean + SEM. *, **, and *** indicate *p* < 0.05, *p* < 0.01, *p* < 0.001, respectively, when compared to control.

Since edaravone was the drug that stood out as the most efficient in decreasing ROS production, we evaluated its effects in a more detailed way. As observed in Figure 17a, 6 h of treatment was not enough to significantly decrease ROS production in cells submitted to OGD. However, after 24 h (Figure 17b) as well as after 48 h (Figure 17c) of treatment, all edaravone concentrations were able to significantly decrease ROS detection in HT-22 cells submitted to OGD insult. These results reinforce the view that edaravone may protect cells submitted to hypoxic insults, possibly by reducing toxic species production in hypoxia injured HT-22 cells.

## 4. Discussion

Neonatal brain injury due to hypoxia–ischemia is an important health problem and is associated with severe neurologic disabilities. Despite advances in the knowledge about the effects of hypoxia–ischemia events in the brain, clinically approved therapeutic interventions for preterm and neonatal hypoxia–ischemia are scarce. In this way, we aimed to test three repurposed drugs commonly used for brain disease treatments, to see if they could revert or at least attenuate hypoxia–ischemia-induced alterations. We used edaravone, perampanel, and metformin, which have already been approved for the treatment of several pathologies and seem to have interesting properties in oxygen and glucose deprivation, namely hypoxia–ischemia insults.

Firstly, we tested how the hippocampal HT-22 cells and SH-SY5Y cells react to the treatment with edaravone, perampanel, and metformin. Among the panoply of brain cell lines, we chose the HT-22 cell lines because it is an established cell line to study neurotoxicity and also because we aim to study, in the future, the potential beneficial effects of these drugs on the treatment of the well-known impairment of learning and memory processes associated with hypoxia–ischemia events [25], particularly in prematurity [26]. Moreover, and for comparative purposes, we decided to test the same drugs in another brain cell line, namely the neuroblastoma SH-SY5Y cell line, to examine whether the potential effect of these drugs is global or, otherwise, specific to some neuronal cell lines. In this way, we tested if these drugs, in a vast range of concentrations and duration of exposure, were cytotoxic to the HT-22 and SH-SY5Y cell lines.

Not surprisingly, we found that edaravone increased cell viability in both types of cells at all time points compared to the control. Our data corroborate several previous reports that have consistently demonstrated that edaravone increases cell viability, has neuroprotective properties [27,28,29], and is a safe drug, at least for the types of cell lines herein studied. Interestingly, both perampanel and metformin had the capacity to increase cell viability when applied during a short period (6 h) and at low concentrations (0.1–1 µM). However, they had no impact on cell viability at medium concentrations and were cytotoxic at higher concentrations (more than 50 µM) and more prolonged treatments (more than 24 h), particularly for perampanel. Although not as efficient as edaravone, metformin and perampanel also can increase cell viability, which is in line with data from previous studies [30,31]. Although these effects of perampanel were particularly obvious when using the MTT assay, which we have demonstrated to be more precise and sensitive to small changes in both cell lines, they were similar to those obtained with the other viability assays used in the present study.

After we tested the cytotoxicity of the present drugs, we then proceeded to evaluate their potential effects in hypoxia–ischemia conditions. In the present work, we used two cellular models of hypoxia–ischemia, one with a hypoxic atmosphere and another that included the association between a hypoxic atmosphere and glucose deprivation. We found that both hypoxic atmosphere and oxygen–glucose deprivation significantly reduced HT-22 cell viability and induced noticeable alterations in their morphology, which was dependent on the time of exposure, corroborating previous studies that have also used hypoxia [32] and oxygen–glucose deprivation [33] cell models. Interestingly, in the neuroblastoma cell line, the hypoxia conditions increased cell viability, despite inducing small morphological changes. Although, we were expecting that this degree of hypoxia could induce significant alterations, even in neuroblastoma cells. This finding was not completely surprising, since it was previously found that hypoxia could favor neuroblastoma cell line survival and proliferation [34,35,36]. Indeed, this could be explained by the activation of different transcriptional programs driven by hypoxia-inducible factors (HIFs) [37] affecting the cell cycle and proliferative status. The HIFs’ effects are context and cell type dependent. In particular, in tumor cells, the genes regulated by these molecules are highly expressed, allowing adaptation mechanisms [38]. Nevertheless, despite the absence of significant alterations in neuroblastoma cells using the present hypoxia model, we decided to test the efficiency of edaravone, metformin, and perampanel also in these cells to see if these drugs were able to improve cell viability during and after the insults.

Among the drugs used in the present study, edaravone was the most efficient in treating the hypoxia-related effects. Indeed, after using this drug, we observed a significant attenuation of hypoxia and oxygen–glucose deprivation effects, demonstrated by a significant increase in cell viability and by a decrease in ROS production, corroborating previous studies showing the efficacy of this drug in hypoxia in vitro [28,39] as well as in vivo models [40,41,42]. We believe that these results support the effects of edaravone on hippocampal HT-22 cells under hypoxic conditions, showing that this drug is very efficient in attenuating the impact of hypoxia in hippocampal cells. Our data also lend support to previous studies showing that edaravone attenuates hypoxia-induced hippocampal damage and cognitive impairment [40]. One mechanism that could explain edaravone’s protective effects on HT-22 cells, in hypoxic conditions, is its free radical scavenging activity [10]. This not only suggests that edaravone activates neuroprotective mechanisms against OGD insults, but also highlights the role of ROS in brain injuries associated with hypoxia–ischemia. Indeed, previous studies have already demonstrated that edaravone attenuates hypoxia-induced neuronal damage via ROS scavenging and upregulation of CREB phosphorylation [40], as well as by reducing apoptotic events and inhibiting hypoxia-inducible factor-1α and cleaved caspase-3 protein expression [41]. In this way, it is tempting to speculate that the alterations of the neurogenic process that we have found in a hypoxia–ischemia in vivo model of previous work, of our group [43], it may be, also, ascribed to changes in these pathways.

Relative to perampanel, an antagonist of AMPA receptors, we have found that this drug, when in low concentrations, exerts beneficial effects against both hypoxia and OGD insults. Whereas in higher concentrations, it decreases cell viability and increases ROS production. Thus, it seems that when in low doses, perampanel may protect neuronal cells from hypoxia and OGD, potentially due to its capability of reducing neuronal overexcitation [13], necroptosis, and neuroinflammatory events [30]. However, it seems that to be efficient, perampanel needs to be administered in low concentrations and simultaneously with hypoxic events. Interestingly, in the present experiment, perampanel was the only drug that increased the viability of neuroblastoma SH-SY5Y cells in normal as well as under hypoxia conditions. However, when in high concentrations, perampanel was not efficient in our hypoxia models, probably because higher concentrations alter normal cell functions and basal excitatory transmission, as previously described [44].

Finally, data obtained in this study indicate that metformin is ineffective in attenuating hypoxic events. This work focused on the insults and treatments in the first phase of the hypoxia–ischemia evolving process. We did not address the reperfusion phase when oxygen and glucose are restored, and inflammatory events are thought to be the major cause of injury. It may be due to this reason that metformin did not show noteworthy results since it is recognized as an anti-inflammatory drug. Indeed, this assumption is partially supported by our finding that metformin was more effective after 48 h of oxygen glucose deprivation than after 6 h of treatment.

By establishing a hypoxia–ischemia model in neuron-like cells, the doors are open to study drugs or compounds that may be able to attenuate the resulting damage and give light on future therapeutic options for hypoxia–ischemia brain injuries. In summary, this work allowed us to demonstrate that edaravone, perampanel, and metformin repurposed drugs are of interest in the early stages of hypoxia–ischemia brain injuries, such as encephalopathy of prematurity. In addition, it also confirmed that ROS production and overexcitation play an important role in the development of the injury. More studies must be performed, mostly in vivo, to confirm our results in a more translational analysis. Furthermore, these three drugs need to be further studied to better understand their mechanism of action and test if their mechanisms of action do not interfere with normal cell function.

## Data Availability

Not applicable.

## References

[1] Tetorou K., Sisa C., Iqbal A., Dhillon K., Hristova M. (2021). Current Therapies for Neonatal Hypoxic–Ischaemic and Infection-Sensitised Hypoxic–Ischaemic Brain Damage. Front. Synaptic Neurosci..

[2] Rocha-Ferreira E., Hristova M. (2016). Plasticity in the Neonatal Brain following Hypoxic-Ischaemic Injury. Neural Plast..

[3] Wang Q., Lv H., Lu L., Ren P., Li L. (2019). Neonatal hypoxic–ischemic encephalopathy: Emerging therapeutic strategies based on pathophysiologic phases of the injury. J. Matern. Neonatal Med..

[4] Ophelders D.R.M.G., Gussenhoven R., Klein L., Jellema R.K., Westerlaken R.J., Hütten M.C., Vermeulen J., Wassink G., Gunn A.J., Wolfs T.G. (2020). Preterm brain injury, antenatal triggers, and therapeutics: Timing is key. Cells.

[5] Distefano G., Praticò A.D. (2010). Actualities on molecular pathogenesis and repairing processes of cerebral damage in perinatal hypoxic-ischemic encephalopathy. Ital. J. Pediatr..

[6] Meldrum B.S. (2000). Glutamate as a Neurotransmitter in the Brain: Review of Physiology and Pathology. J. Nutr..

[7] Dixon B.J., Reis C., Ho W.M., Tang J., Zhang J.H. (2015). Neuroprotective Strategies after Neonatal Hypoxic Ischemic Encephalopathy. Int. J. Mol. Sci..

[8] Juul S.E., Ferriero D.M. (2014). Pharmacologic Neuroprotective Strategies in Neonatal Brain Injury. Clin. Perinatol..

[9] Kikuchi K., Uchikado H., Miyagi N., Morimoto Y., Ito T., Tancharoen S., Miura N., Miyata K., Sakamoto R., Kikuchi C. (2011). Beyond neurological disease: New targets for edaravone (Review). Int. J. Mol. Med..

[10] Kawasaki H., Ito Y., Kitabayashi C., Tanaka A., Nishioka R., Yamazato M., Ishizawa K., Nagai T., Hirayama M., Takahashi K. (2020). Effects of Edaravone on Nitric Oxide, Hydroxyl Radicals and Neuronal Nitric Oxide Synthase During Cerebral Ischemia and Reperfusion in Mice. J. Stroke Cerebrovasc. Dis..

[11] Yoshino H. (2019). Edaravone for the treatment of amyotrophic lateral sclerosis. Expert Rev. Neurother..

[12] Shakkour Z., Issa H., Ismail H., Ashekyan O., Habashy K.J., Nasrallah L., Jourdi H., Hamade E., Mondello S., Sabra M. (2021). Drug Repurposing: Promises of Edaravone Target Drug in Traumatic Brain Injury. Curr. Med. Chem..

[13] Lattanzi S., Striano P. (2019). The impact of perampanel and targeting AMPA transmission on anti-seizure drug discovery. Expert Opin. Drug Discov..

[14] Besag F.M., Patsalos P.N. (2016). Clinical efficacy of perampanel for partial-onset and primary generalized tonic-clonic seizures. Neuropsychiatr. Dis. Treat..

[15] Zhou J., Massey S., Story D., Li L. (2018). Metformin: An Old Drug with New Applications. Int. J. Mol. Sci..

[16] Venna V.R., Li J., Hammond M.D., Mancini N.S., McCullough L.D. (2014). Chronic metformin treatment improves post-stroke angiogenesis and recovery after experimental stroke. Eur. J. Neurosci..

[17] Wang Y.-W., He S.-J., Feng X., Cheng J., Luo Y.-T., Tian L., Huang Q. (2017). Metformin: A review of its potential indications. Drug Des. Dev. Ther..

[18] Wang G.-H., Jiang Z.-L., Li Y.-C., Li X., Shi H., Gao Y.-Q., Vosler P.S., Chen J. (2011). Free-Radical Scavenger Edaravone Treatment Confers Neuroprotection Against Traumatic Brain Injury in Rats. J. Neurotrauma.

[19] Yu H., Wu Z., Wang X., Gao C., Liu R., Kang F., Dai M. (2020). Protective effects of combined treatment with mild hypothermia and edaravone against cerebral ischemia/reperfusion injury via oxidative stress and Nrf2 pathway regulation. Int. J. Oncol..

[20] Yuan Y., Zha H., Rangarajan P., Ling E.-A., Wu C. (2014). Anti-inflammatory effects of Edaravone and Scutellarin in activated microglia in experimentally induced ischemia injury in rats and in BV-2 microglia. BMC Neurosci..

[21] Qi X., Okuma Y., Hosoi T., Nomura Y. (2004). Edaravone Protects against Hypoxia/Ischemia-Induced Endoplasmic Reticulum Dysfunction. J. Pharmacol. Exp. Ther..

[22] Wu D., Yotnda P. (2011). Induction and Testing of Hypoxia in Cell Culture. J. Vis. Exp..

[23] Salvador E., Burek M., Förster C.Y. (2015). Stretch and/or oxygen glucose deprivation (OGD) in an in vitro traumatic brain injury (TBI) model induces calcium alteration and inflammatory cascade. Front. Cell. Neurosci..

[24] Larsson P., Engqvist H., Biermann J., Rönnerman E.W., Forssell-Aronsson E., Kovács A., Karlsson P., Helou K., Parris T.Z. (2020). Optimization of cell viability assays to improve replicability and reproducibility of cancer drug sensitivity screens. Sci. Rep..

[25] Ikeda T., Mishima K., Yoshikawa T., Iwasaki K., Fujiwara M., Xia Y.X., Ikenoue T. (2001). Selective and long-term learning impairment following neonatal hypoxic-ischemic brain insult in rats. Behav. Brain Res..

[26] Omizzolo C., Scratch S.E., Stargatt R., Kidokoro H., Thompson D.K., Lee K.J., Cheong J., Neil J., Inder T.E., Doyle L.W. (2013). Neonatal brain abnormalities and memory and learning outcomes at 7 years in children born very preterm. Memory.

[27] Zhao Z.-Y., Luan P., Huang S.-X., Xiao S.-H., Zhao J., Zhang B., Gu B.-B., Pi R.-B., Liu J. (2013). Edaravone Protects HT22 Neurons from H_2_O_2_-induced Apoptosis by Inhibiting the MAPK Signaling Pathway. CNS Neurosci. Ther..

[28] Guo Z., Wu H.-T., Li X.-X., Yu Y., Gu R.-Z., Lan R., Qin X.-Y. (2020). Edaravone protects rat astrocytes from oxidative or neurotoxic inflammatory insults by restoring Akt/Bcl-2/Caspase-3 signaling axis. IBRO Rep..

[29] Cha S.J., Kim K. (2022). Effects of the Edaravone, a Drug Approved for the Treatment of Amyotrophic Lateral Sclerosis, on Mitochondrial Function and Neuroprotection. Antioxidants.

[30] Yang L., Wang Y., Zhang C., Chen T., Cheng H. (2021). Perampanel, an AMPAR antagonist, alleviates experimental intracerebral hemorrhage-induced brain injury via necroptosis and neuroinflammation. Mol. Med. Rep..

[31] Ge J., Huang Y., Zhang Y., Liu L., Gu T., Liu X., Yao L., Cai M., Sun J., Song J. (2020). Metformin Inhibits Propofol-Induced Apoptosis of Mouse Hippocampal Neurons HT-22 Through Downregulating Cav-1. Drug Des. Dev. Ther..

[32] Chhunchha B., Fatma N., Kubo E., Rai P., Singh S.P., Singh D.P. (2013). Curcumin abates hypoxia-induced oxidative stress based-ER stress-mediated cell death in mouse hippocampal cells (HT22) by controlling Prdx6 and NF-kappaB regulation. Am. J. Physiol. Cell Physiol..

[33] Cai B., Li W., Mao X., Winters A., Ryou M.-G., Liu R., Greenberg D.A., Wang N., Jin K., Yang S.-H. (2016). Neuroglobin Overexpression Inhibits AMPK Signaling and Promotes Cell Anabolism. Mol. Neurobiol..

[34] Jögi A., Øra I., Nilsson H., Lindeheim Å., Makino Y., Poellinger L., Axelson H., Påhlman S. (2002). Hypoxia alters gene expression in human neuroblastoma cells toward an immature and neural crest-like phenotype. Proc. Natl. Acad. Sci. USA.

[35] Påhlman S., Mohlin S. (2018). Hypoxia and hypoxia-inducible factors in neuroblastoma. Cell Tissue Res..

[36] Hubbi M.E., Semenza G.L. (2015). Regulation of cell proliferation by hypoxia-inducible factors. Am. J. Physiol. Cell Physiol..

[37] Hussein D., Estlin E.J., Dive C., Makin G.W. (2006). Chronic hypoxia promotes hypoxia-inducible factor-1α–dependent resistance to etoposide and vincristine in neuroblastoma cells. Mol. Cancer Ther..

[38] Al Tameemi W., Dale T.P., Al-Jumaily R.M.K., Forsyth N.R. (2019). Hypoxia-Modified Cancer Cell Metabolism. Front. Cell Dev. Biol..

[39] Cao B., Chai C., Zhao S. (2015). Protective effect of Edaravone against hypoxia-induced cytotoxicity in osteoblasts MC3T3-E1 cells. IUBMB Life.

[40] Ling J., Yu Q., Li Y., Yuan X., Wang X., Liu W., Guo T., Duan Y., Li L. (2020). Edaravone Improves Intermittent Hypoxia-Induced Cognitive Impairment and Hippocampal Damage in Rats. Biol. Pharm. Bull..

[41] Lei S., Zhang P., Li W., Gao M., He X., Zheng J., Li X., Wang X., Wang N., Zhang J. (2014). Pre- and posttreatment with edaravone protects CA1 hippocampus and enhances neurogenesis in the subgranular zone of dentate gyrus after transient global cerebral ischemia in rats. ASN Neuro.

[42] Li C., Mo Z., Lei J., Li H., Fu R., Huang Y., Luo S., Zhang L. (2018). Edaravone attenuates neuronal apoptosis in hypoxic-ischemic brain damage rat model via suppression of TRAIL signaling pathway. Int. J. Biochem. Cell Biol..

[43] Rocha R., Andrade L., Alves T., Sá S., Pereira P.A., Madeira M.D., Cardoso A. (2021). Behavioral and brain morphological analysis of non-inflammatory and inflammatory rat models of preterm brain injury. Neurobiol. Learn. Mem..

[44] Mazzocchetti P., Mancini A., Sciaccaluga M., Megaro A., Bellingacci L., Di Filippo M., Cesarini E.N., Romoli M., Carrano N., Gardoni F. (2020). Low doses of Perampanel protect striatal and hippocampal neurons against in vitro ischemia by reversing the ischemia-induced alteration of AMPA receptor subunit composition. Neurobiol. Dis..

